# Dynamics of Cortical Degeneration Over a Decade in Huntington’s Disease

**DOI:** 10.1016/j.biopsych.2020.11.009

**Published:** 2021-04-15

**Authors:** Eileanoir B. Johnson, Gabriel Ziegler, William Penny, Geraint Rees, Sarah J. Tabrizi, Rachael I. Scahill, Sarah Gregory

**Affiliations:** a Huntington's Disease Centre, Department of Neurodegenerative Disease, UCL Queen Square Institute of Neurology, University College London, London, United Kingdom; b Wellcome Centre for Human Neuroimaging, UCL Queen Square Institute of Neurology, University College London, London, United Kingdom; c Dementia Research Institute at University College London, London, United Kingdom; d School of Psychology, University of East Anglia, Norwich, United Kingdom; e Institute of Cognitive Neurology and Dementia Research, Otto-von-Guericke-University Magdeburg, Magdeburg, Germany; f German Center for Neurodegenerative Diseases, Magdeburg, Germany

**Keywords:** Atrophy, Cortex, Huntington’s disease, Imaging, Longitudinal, Subcortex

## Abstract

**Background:**

Characterizing changing brain structure in neurodegeneration is fundamental to understanding long-term effects of pathology and ultimately providing therapeutic targets. It is well established that Huntington’s disease (HD) gene carriers undergo progressive brain changes during the course of disease, yet the long-term trajectory of cortical atrophy is not well defined. Given that genetic therapies currently tested in HD are primarily expected to target the cortex, understanding atrophy across this region is essential.

**Methods:**

Capitalizing on a unique longitudinal dataset with a minimum of 3 and maximum of 7 brain scans from 49 HD gene carriers and 49 age-matched control subjects, we implemented a novel dynamical systems approach to infer patterns of regional neurodegeneration over 10 years. We use Bayesian hierarchical modeling to map participant- and group-level trajectories of atrophy spatially and temporally, additionally relating atrophy to the genetic marker of HD (CAG-repeat length) and motor and cognitive symptoms.

**Results:**

We show, for the first time, that neurodegenerative changes exhibit complex temporal dynamics with substantial regional variation around the point of clinical diagnosis. Although widespread group differences were seen across the cortex, the occipital and parietal regions undergo the greatest rate of cortical atrophy. We have established links between atrophy and genetic markers of HD while demonstrating that specific cortical changes predict decline in motor and cognitive performance.

**Conclusions:**

HD gene carriers display regional variability in the spatial pattern of cortical atrophy, which relates to genetic factors and motor and cognitive symptoms. Our findings indicate a complex pattern of neuronal loss, which enables greater characterization of HD progression.

Characterizing the temporal trajectory of cortical atrophy is important for the development of mechanistic theories of neurodegeneration. Uncovering the cortical areas that undergo atrophy along with the associated atrophy rates during different phases of neurodegeneration can provide insights into the biological underpinnings of neurodegenerative disease. Until now, characterizing the dynamic patterns of brain change has been limited by the lack of suitable modeling frameworks and cohort data with extensive time points (1), resulting in limited knowledge of the nature of long-term brain changes. Here, we use a Huntington’s disease (HD) cohort to validate a novel method of quantifying longitudinal trajectories of neurodegeneration over a large number of time points. HD is an ideal neurodegenerative condition in which to validate this technique because a definitive genetic test can identify the condition long before symptom onset and clinical diagnosis, and it is a well-phenotyped progressive neurodegenerative disease (2).

Despite detailed knowledge of the genetic cause and symptoms of HD, the underlying cellular mechanisms and pathophysiology are not well understood. There is robust evidence that striatal degeneration begins over a decade before symptom onset (3,4) and continues at a constant rate (5, 6, 7, 8, 9). By early manifest disease, cortical atrophy appears to have occurred (4), but the ongoing process of gray matter (GM) degeneration in HD has not been studied. There is evidence of increasing white matter disorganization during this period (10, 11, 12, 13), but there have been conflicting findings regarding the cortex, with regional cortical change only described between two time points over short intervals and via restrictive analysis techniques (6,14, 15, 16, 17, 18, 19) such as regression models. Because HD is a slowly progressive disease, these studies ultimately fail to capture the nature or extent of cortical change. It has proven challenging to understand the neural bases of heterogeneity in HD onset and symptom progression, despite an apparent association between postmortem cortical degeneration and symptomatology before death (20). With the advent of genetic therapies targeting the cortex (21), a greater understanding of long-term cortical processes and their impact on clinical progression is essential.

Here, we apply a novel modeling technique to map volumetric brain changes and the associated clinical changes over 10 years in a large group of HD gene carriers. This technique, which capitalizes on Bayesian hierarchical modeling, offers a powerful approach to defining change across numerous data points. Furthermore, it can be used to test for causal interactions between changes in different brain regions. The approach constructs individual participant-level dynamic models of atrophy, which are used to identify groupwise trajectories of disease progression both spatially and temporally (22). By specifying temporal progression of cortical atrophy within all brain regions simultaneously (23), both total atrophy and, uniquely, rates of atrophy over multiple time points can be understood. These new insights reveal not only where change occurs but, for the first time, how the pace of regional neurodegeneration varies across the cortex. Importantly, the model can examine the influence of external factors on brain changes (e.g., genetic components), identify causal patterns of interregional interactions, and predict behavioral scores from regional atrophy.

We applied this technique (22, 23, 24) to compare cortical brain changes in a cohort of HD gene carriers during a 10-year period surrounding onset of motor symptoms with an age-matched control group from the multisite, longitudinal TRACK-HD and TrackOn-HD studies (4,6,7,14,25). Motor onset is a critical period in HD progression, and it is used as a proxy for clinically diagnosed disease onset. During this period, the increasing prevalence of motor symptoms results in increased clinical interventions and greater disruption to everyday functioning and mental well-being (26). The nature of these motor symptoms suggests a breakdown of the motor network; however, to understand the progression in motor symptoms, long-term mapping of degeneration trajectories alongside clinical measures is essential.

We analyzed up to 7 individual annual magnetic resonance imaging (MRI) scans per participant plus evaluations of motor and cognitive performance for the HD group, focusing on volumetric measures from widespread cortical (and subcortical) brain regions using a protocol optimized for this cohort (27). We investigated in which regions the HD patients showed lower volume at the point of diagnosis, and how the rates of atrophy varied across the cortex during this period compared with the control participants. We predicted that subcortical atrophy would show the greatest degeneration (28), with regions of the frontal, parietal, and occipital cortices also expected to show atrophy. We hypothesized that the HD participants with higher CAG-repeat lengths (the genetic cause of HD) would undergo greater atrophy.

## Methods and Materials

### Participants

The study participants were from the TRACK-HD and TrackOn-HD cohorts (4,25). Pre-HD participants from both cohorts who subsequently transitioned to manifest HD (converters) during the data collection period were included and were used to create a group experiencing a similar stage of disease progression. The control participants were selected from the same datasets to match the HD group as closely as possible for age, gender, site, and number of visits (Supplemental Methods and Materials). The study was approved by the local ethics committees, and written informed consent was obtained from each participant according to the Declaration of Helsinki.

To increase the homogeneity of disease progression and define a comparable progression time variable, the data were realigned to consolidate the year of motor conversion across all participants (Figure S1). The first year of diagnostic confidence score = 4 was designated as the year of conversion (time point 0), and each year before conversion was labeled as year −1, −2, −3, and so on. Every year after conversion was labeled as year 1, 2, 3, and so on. The individual variability of changes beyond the synchronizing event of motor diagnosis was accounted for during modeling. Every HD participant was matched with a control participant, who was aligned with “time point 0,” the point at which his or her age matched the corresponding HD participant. Participants had a minimum of 3 and maximum of 7 time points (HD mean = 5.84 scans, SD = 1.63; control mean = 6.06 scans, SD = 1.45).

The Unified Huntington’s Disease Rating Scale Total Motor Score (TMS) was used to approximate clinical motor progression (29) (Supplemental Methods and Materials). The Symbol Digit Modalities Test (SDMT) was included as a measure of cognitive progression (30). The SDMT, a cognitive task designed to measure visual processing and psychomotor speed, has been established as the most reliable and sensitive cognitive measure for detecting change in premanifest and manifest HD (31,32) and related to HD progression (7). Both scores were inverted and rescaled to [0,100] to the min/max observation in the HD sample with an increase indicating worsening symptoms.

### MRI Data Acquisition

We acquired T1-weighted scans from four 3T scanners with acquisition protocols that were the same for both studies (Supplemental Methods and Materials).

### Longitudinal Image Processing

A longitudinal within-participant registration pipeline from SPM12 was used to create an average image for each participant (33); this was parcellated into 138 regions using MALP-EM software (Biomedical Image Analysis Group, London, United Kingdom), a fully automated segmentation tool (34) validated for use in HD (27). Each average segmented region was multiplied by Jacobian deformation maps (derived from registration) to create a volumetric map for each region for each time point (see Supplemental Methods and Materials). All segmentations underwent visual quality control. One dataset failed quality control owing to segmentation errors.

To reduce noise within small cortical regions, we combined the segmentations into 55 larger regions based on spatial localization and visual inspection (Table S1). We included 50 cortical regions (25 bilateral pairs), 4 subcortical regions (bilateral caudate and putamen), and 1 global white matter region. To facilitate clear across-region comparisons, we analyzed regional brain volumes (%) relative to the sample overall mean volume at the time point of motor diagnosis (set to 100%).

### Hierarchical Disease Progression Model Using Bayesian Inference

**Figure 1:**
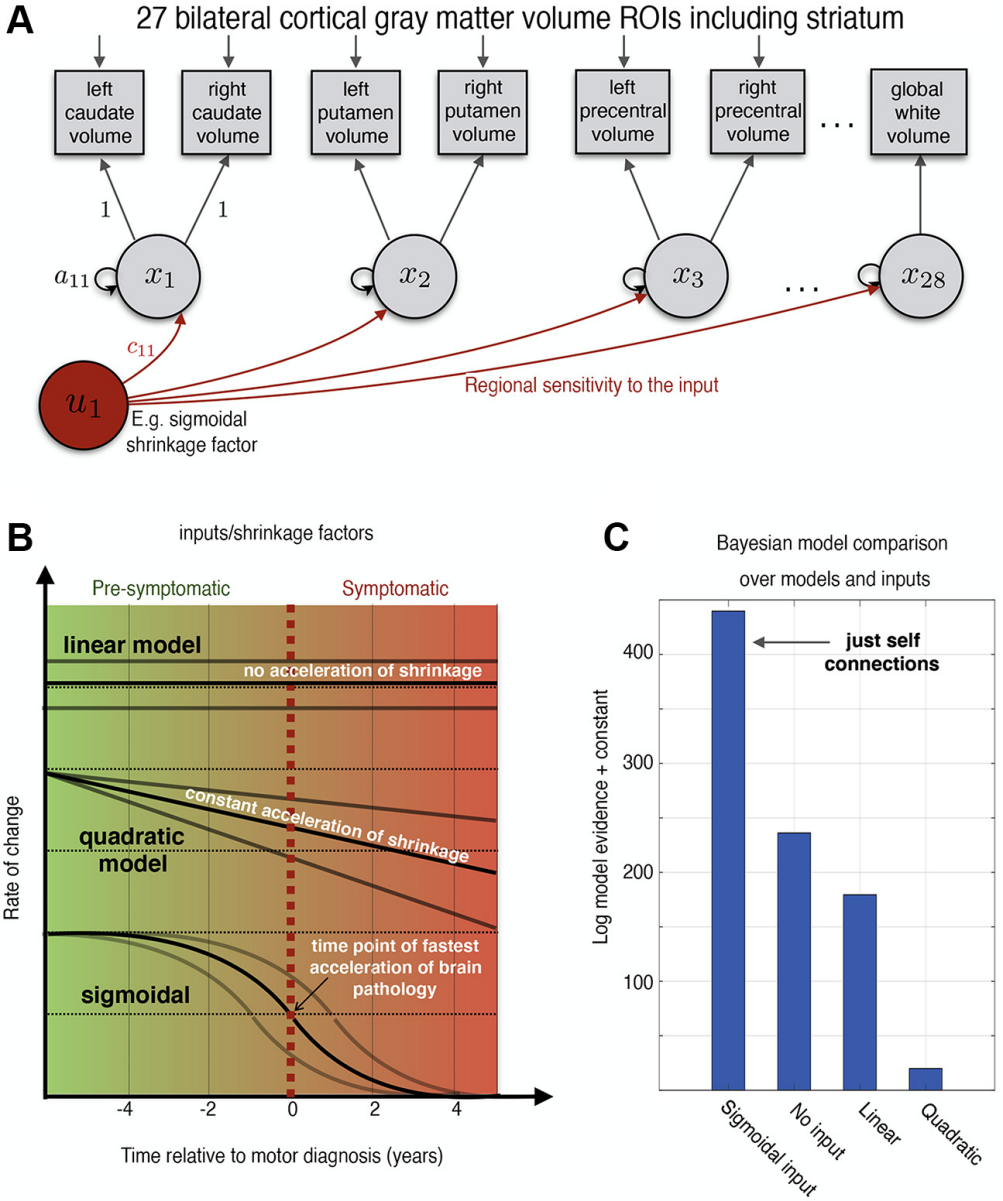
Model illustration and Bayesian model comparison. **(A)** An illustration of participant-level models. The model is defined by a system describing changes in observed volumes (squares) using 28 regional latent state variables (circles) during the period studied. The states are negatively self-connected causing region-specific decline (atrophy) of volume. **(B)** Illustration of system inputs (red circle) that were explored causing different forms of acceleration of pathology during the transition from presymptomatic to symptomatic disease phase. In case of presence of nonlinearities, the rate of change (velocity) of progression might not be constant (top) but changes linearly with progression time (middle) or transition smoothly following a sigmoidal shape (bottom). **(C)** Approximate model evidence of multiple models compared. All models compared were hierarchical, with subject-level and a group-level to describe commonalities and control/Huntington’s disease differences, covariates, and confounds. ROIs, regions of interest.

Hierarchical (multilevel) modeling is an increasingly popular approach for modeling longitudinal data, outperforming classic regression in its predictive accuracy (35). Here we used a previously established framework for dynamic modeling of longitudinal structural MRI (23) using Bayesian inference (22) (Figure 1). We summarize the relevant components of the model here (and in the Supplemental Methods and Materials) and refer the mathematically interested reader to a more technical introduction (23,36,37). The dynamical system used for modeling brain changes is generally described via state model

$$\frac{dx}{dt}\left(t\right)\;=\;Ax\left(t\right)\;+\;Cu\left(t,\;\theta_{u}\right)$$

and observational (or measurement) model

$$y\left(t\right)\;=\;g\left(x\left(t\right),\;\theta_{g}\right)\;+\;\varepsilon$$

with multivariate observations *y*(*t*), state variables *x*(*t*), system inputs *u*(*t*), connectivity parameter matrix *A*, regional sensitivity parameter to inputs *C*, and residuals *ε.* More specifically, the state equation models the temporal progression of the state vector *x*(*t*), referring to 27 bilateral volumes (25 cortical regions, caudate, and putamen) and one global white matter volume over 10-year periods (*t* = −6, …, 5 years relative to diagnosis).

The progression of states is influenced by both endogenous dynamics *Ax*(*t*) and external time-varying inputs *u*(*t*,*θ*_*u*_), with optional input parameters *θ*_*u*_. The endogenous dynamics of the HD model were restricted to regional self-connections, which can be interpreted as region-specific atrophy (or decay) rates resulting in approximately linear volume loss over the course of progression. We assumed bilateral symmetry of disease progression across hemispheres; thus, the same state variable describes evolution of volumes in both corresponding bilateral GM regions of interest (via a linear observational model *g* that averages both hemispheres). In summary, the generative model makes predictions for 55 brain regions using 28 dynamical state variables describing region-specific volume progression during the decade around disease onset. Three to 7 available scans per person were used to optimize both individual- and group-level model parameters in a two-stage procedure.

As outlined in Zeidman *et al.* (36,37), the Bayesian modeling framework enables comparison of alternative individual-level and group-level models that implement hypotheses about brain data. However, because our goal was regional mapping of a priori unknown structural disease progression dynamics, we applied this approach in a more exploratory way. We compared four conventional and novel individual first-level state models that might be useful to describe volume progression toward HD (Figure 1B): 1) a linear model—that is, a constant rate of atrophy [*A* = 0, *C* ≠ 0, *u*(*t*) = *c*_0_]; 2) a quadratic model—that is, accelerated change [*A* = 0, *C* ≠ 0, *u*(*t*) = *c*_0_ *+ c*_1_*t*]; 3) a simple dynamic model without inputs (i.e., *A* ≠ 0, *C* = 0); and 4) a more complex dynamic model with sigmoidal input *u* (Supplemental Methods and Materials). The choice of a sigmoidal input was motivated by its wide use in the context of hypothetical and data-driven models of other neurodegenerative diseases (38, 39, 40, 41). Each of these first-level models (1 to 4) was estimated for each participant and was inverted using variational Laplace methods (24).

Multilevel modeling increases power for detecting group-level effects by modeling differences and uncertainty in first-level parameters while accounting for the differing number of visits. First-level models for each participant were embedded in a second-level model to estimate groupwise brain change, the advantages of which are discussed in the Supplemental Methods and Materials but pertain to statistical efficiency and mitigating risk of overfitting. Bayesian hierarchical models for each of the four first-level models (1 to 4) were estimated using parametric empirical Bayes (PEB) (22,36), incorporating a second-level design matrix with overall sample mean, diagnostic group difference, and covariates including CAG-repeat length, gender, age (at motor diagnosis), total intracranial volume, and site (age orthogonalized with respect to CAG due to high correlation).

Bayesian model selection was then used to compare statistical evidence for each of models 1 to 4 at the whole-sample level. Bayesian model selection optimizes model fit while penalizing complexity and is appropriate for use in highly parameterized hierarchical disease progression models (42). Of the four models, evidence was highest for the simple dynamic model (state equation *dx*/*dt* = *Ax*) using no inputs (Figure 1C). Consequently, the optimal model was found to be comparably parsimonious.

Notably, we followed recommendations from the American Statistical Association (43) and used Bayesian inference in our main analysis (Supplemental Methods and Materials), although some *p-*value hypothesis tests are reported for demographics. For all group-level model parameters, such as group difference of the initial state (volume at diagnosis) or (log) decay rate, we present (Bayesian) posteriors mean ± SD. Moreover, as suggested by Zeidman *et al.* (36), we used Bayesian model reduction and averaging to reduce numbers of parameters and threshold parameters of the winning model based on free energy. This involved, for each second-level parameter *j*, performing a Bayesian model comparison of the hierarchical PEB model with parameter *j* switched on (free to vary) versus the equivalent PEB model with parameter *j* switched off (fixed at its prior expectation of 0). The difference in evidence can then be converted to a posterior probability. The results focus on parameters from the Bayesian model reduction and averaging that exceed the posterior probability threshold of .95.

Next, we extended the observational model to investigate the possible interregional dynamics of morphometry during HD progression and additionally to predict motor and cognitive symptom scores (Supplemental Methods and Materials). For further validation of the hierarchical dynamical model we assessed its predictive validity to determine the clinical significance of model parameters using leave-one-out cross-validation. The above winning model was fitted to all but one participant, and covariates (group membership HD vs. control and CAG) for the left-out participant were predicted. This was repeated with each participant left out, and the accuracy of the prediction was recorded (Figure S2). The predictive validity when using the model parameters to predict individual group membership was found to be very high, with 97 of 98 subjects correctly assigned using their posterior probabilities (estimated and true group variable correlate *r* = .9). When predicting CAG, the estimated and true values correlated *r* = .41.

### Data and Code Availability

Requests for access to TRACK-HD and TrackOn-HD data should be made via the CHDI Foundation. Links to custom-made scripts and the synthetic example dataset demonstrating dynamic modeling of longitudinal HD data can be provided upon request to the corresponding authors (Supplemental Methods and Materials).

## Results

### Sample

We analyzed longitudinal data from 49 HD gene carriers with 3 to 7 individual annual scans (mean = 5.84, SD = 1.63) over a follow-up time of 2 to 6 years (mean = 5.94, SD = 1.62), and 49 control participants with 3 to 7 annual scans (mean = 6.08, SD = 1.45) (Figure S1). Thirty HD participants and 33 control participants had 7 annual scans. The demographics are shown in Table 1. There was no significant group difference in age. As expected, the HD gene carriers with longer CAG-repeat length had an earlier clinical diagnosis (*r* = −.85, *p* < .001).

**Table 1:** Participant Demographics

	HD	Control
Age, Years	44.59 (9.28) [28.65–66.00]	44.51 (9.04) [28.85–66.06]
Female	27 (55.10%)	30 (61%)
CAG	43.67 (2.77) [39.00–50.00]	NA
Site		
Leiden	22 (44.90%)	14 (28.57%)
London	10 (20.40%)	10 (20.40%)
Paris	10 (20.40%)	14 (28.57%)
Vancouver	7 (14.29%)	11 (22.45%)

Values represent mean (SD) [range] or *n* (%).

HD, Huntington’s disease; NA, not applicable.

### Widespread Group Differences Are Found at HD Motor Diagnosis

**Figure 2:**
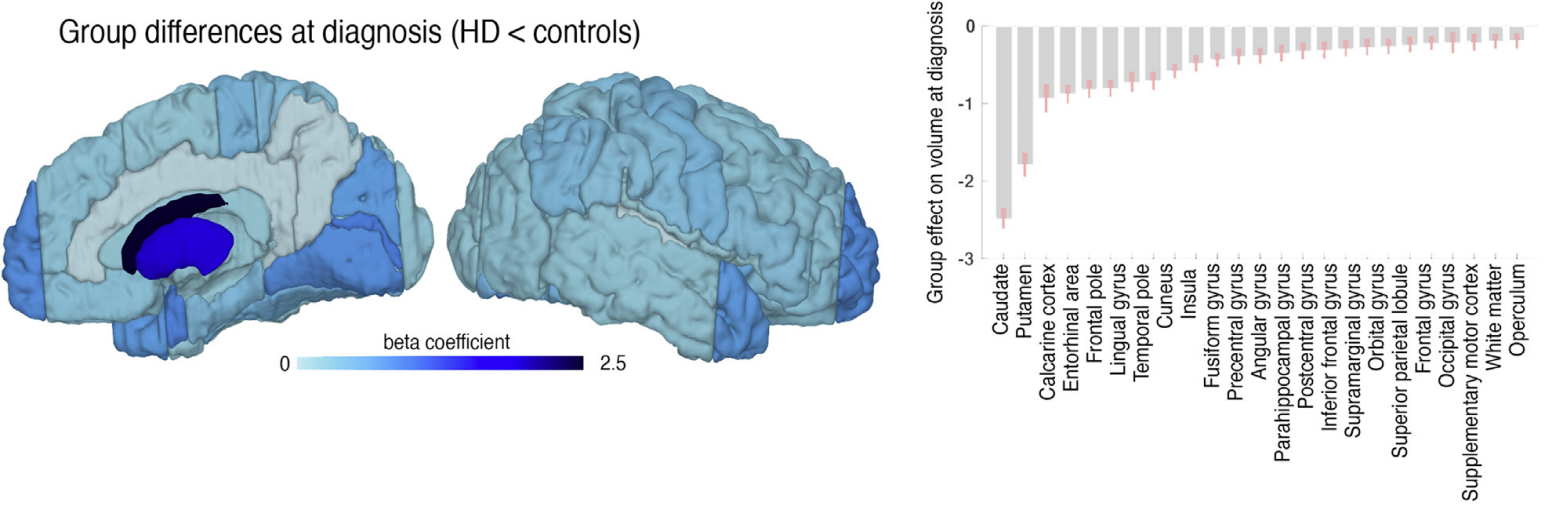
Volume differences between Huntington’s disease (HD) and control subjects at point of motor diagnosis: group differences of volumes (HD < control) at time point of motor diagnosis as predicted by the dynamic disease progression model. Shown are surface projections (left) and bar plots ± SD (right panel) of the group-level regional offset parameters (initial states) at time point 0 obtained from Bayesian model reduction and averaging. White indicates nonsignificant group differences. Only significant regions are shown in the bar plot.

When comparing the HD participants with age-matched control participants at time point of motor diagnosis, we found widespread differences in volume across the brain. As predicted, caudate and putamen showed the largest differences (Figure 2), with regions across all lobes also showing group differences, demonstrating that cortical atrophy is extensive even at this early stage of HD.

### Atrophy Over a Decade Is Variable Across the Cortex

**Figure 3:**
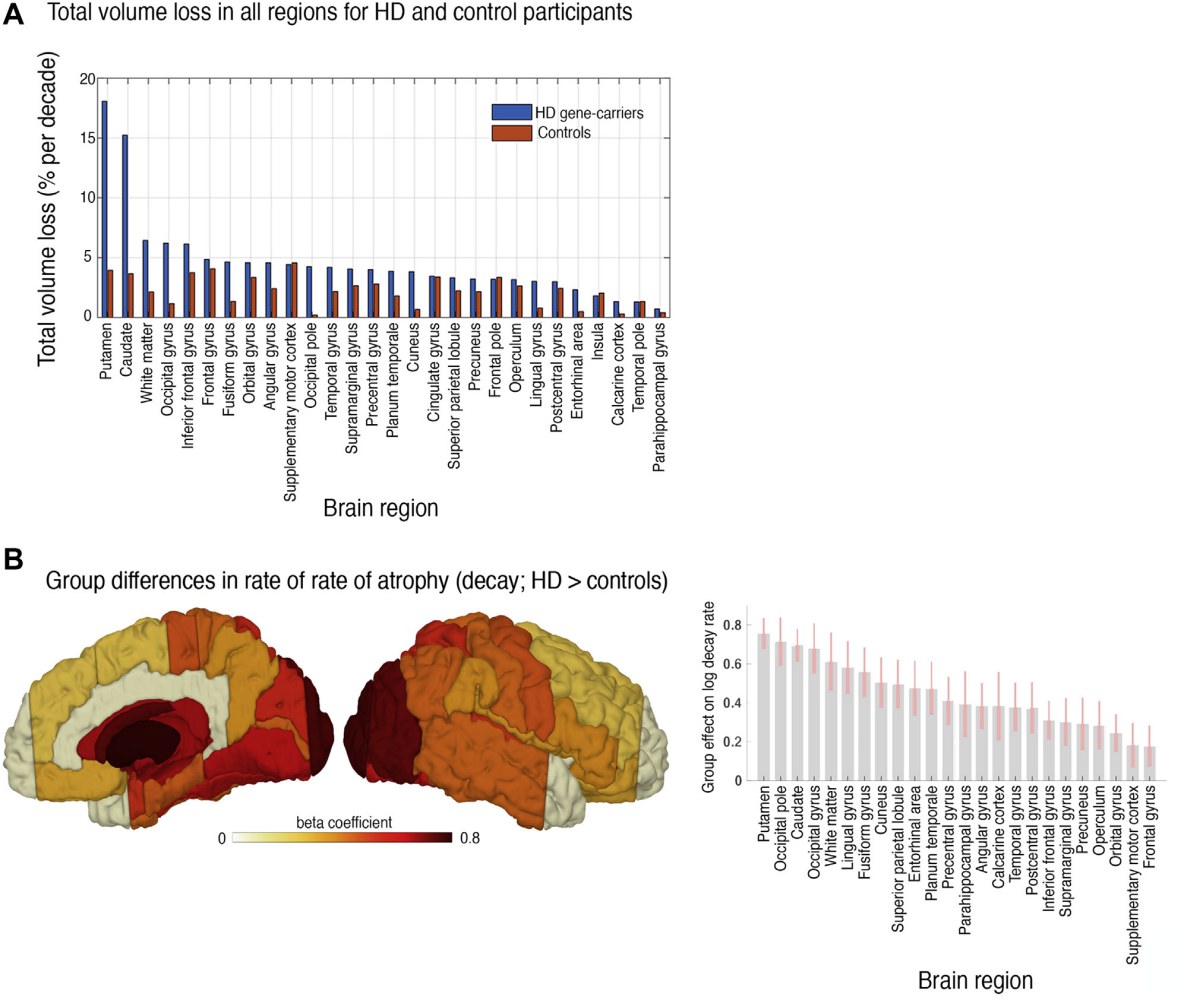
Total brain atrophy during Huntington’s disease (HD) motor conversion and rates of atrophy during HD motor conversion. **(A)** Parameter plot of the overall percent volume loss per region per decade approximated by a linear model. Median total volume loss (in % per decade) is presented using a nonhierarchical model to minimize the influence of priors on group and region-specific rates of change. However, the results were coarsely consistent with the predictions from the dynamical Bayesian hierarchical further presented. **(B)** Significant rate of atrophy in group differences (HD > control) over a decade around HD motor onset. Decay rate refers to self-connection parameters of regional volume states (see Methods and Materials). Both panels use log scale for illustration. All results are group-level estimates based on longitudinal dynamic modeling and account for effects of age, gender, CAG, and confounds.

Over a decade of HD progression, when compared with control subjects, the highest total volume reduction was in striatal regions; the putamen and caudate showed 18.7% and 15.4% loss of baseline volume in HD, respectively, but less than 3% for both regions in control subjects (Figure 3A). The rate of volume loss was higher in widespread cortical areas for HD participants (Figure 3B and Figure S3), particularly the occipital and parietal regions (superior parietal lobule, precentral gyrus). These findings highlight pronounced posterior atrophy during the long-term transition from pre-HD to manifest HD, suggesting a distinct spatiotemporal pattern of change associated with clinical presentation.

### Brain Atrophy Is Related to Genetic Burden in Some Regions

**Figure 4:**
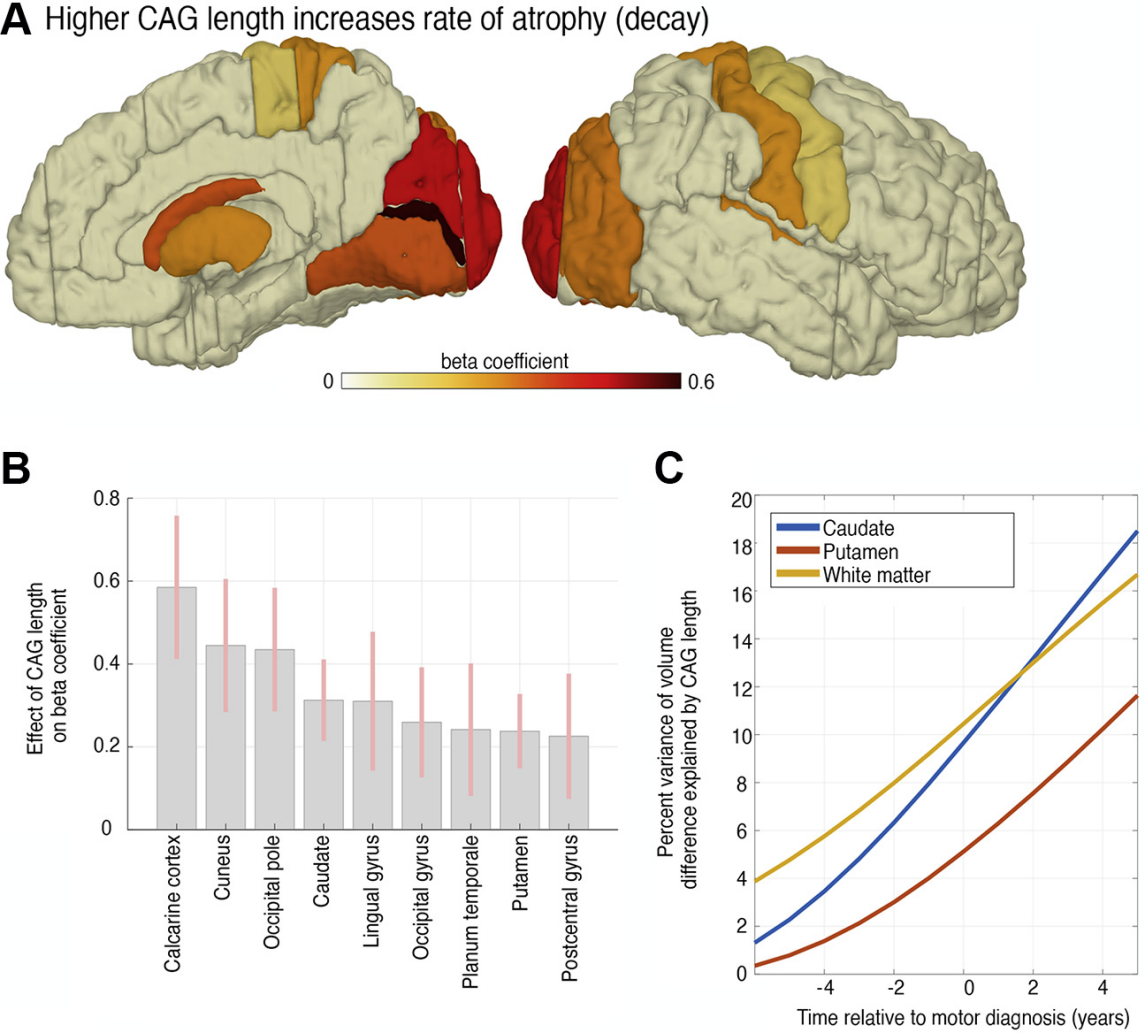
CAG-repeat length related to rate of cortical and striatal atrophy. **(A)** A brain surface projection and **(B)** parameter ± SD that indicates whether individual CAG-repeat length predicts regional rate of atrophy. Participants with higher CAG-repeat length showed an increased rate of atrophy, especially in posterior cortical and striatal areas. White indicates nonsignificant CAG effects. Only significant regions are shown in bar plot. Analysis from Bayesian model average accounted for effects of age, gender, and confounds (see Methods and Materials). **(B)** Proportion of total variance of volume in caudate/putamen/white matter explained (i.e., *R*^2^) by CAG-repeat length. We show *R*^2^ over all time points 6 years before to 5 years after diagnosis. x-axis: disease progression time in years relative to individual motor diagnosis.

We further analyzed the link between CAG-repeat length and atrophy across brain regions. CAG-repeat length predicted the rate of atrophy in occipital, parietal, and striatal regions (Figure 4A), suggesting greater vulnerability of the occipital lobe in particular to increased genetic burden. Moreover, systematic effects of CAG-repeat length on progression were reflected in the increasing variance of atrophy explained by gene differences (Figure 4B).

### No Evidence Is Found for Interregional Progression

To explore potential disease spread within the cortex, we compared models that enabled associations of atrophy dynamics between subcortical-cortical and cortical-cortical regions. More specifically, we included between-region connections to test whether atrophy state in one brain area caused volume change in another (connected) brain area. However, model comparisons revealed highest evidence for models without interregional interactions (Figure S4 and Supplemental Methods and Materials), suggesting that either the pattern of regional atrophy is better described independently or the spread of atrophy during HD progression follows a more complex pattern.

### Cortical Atrophy Can Be Linked With Individual Motor and Cognitive Symptom Changes

**Figure 5:**
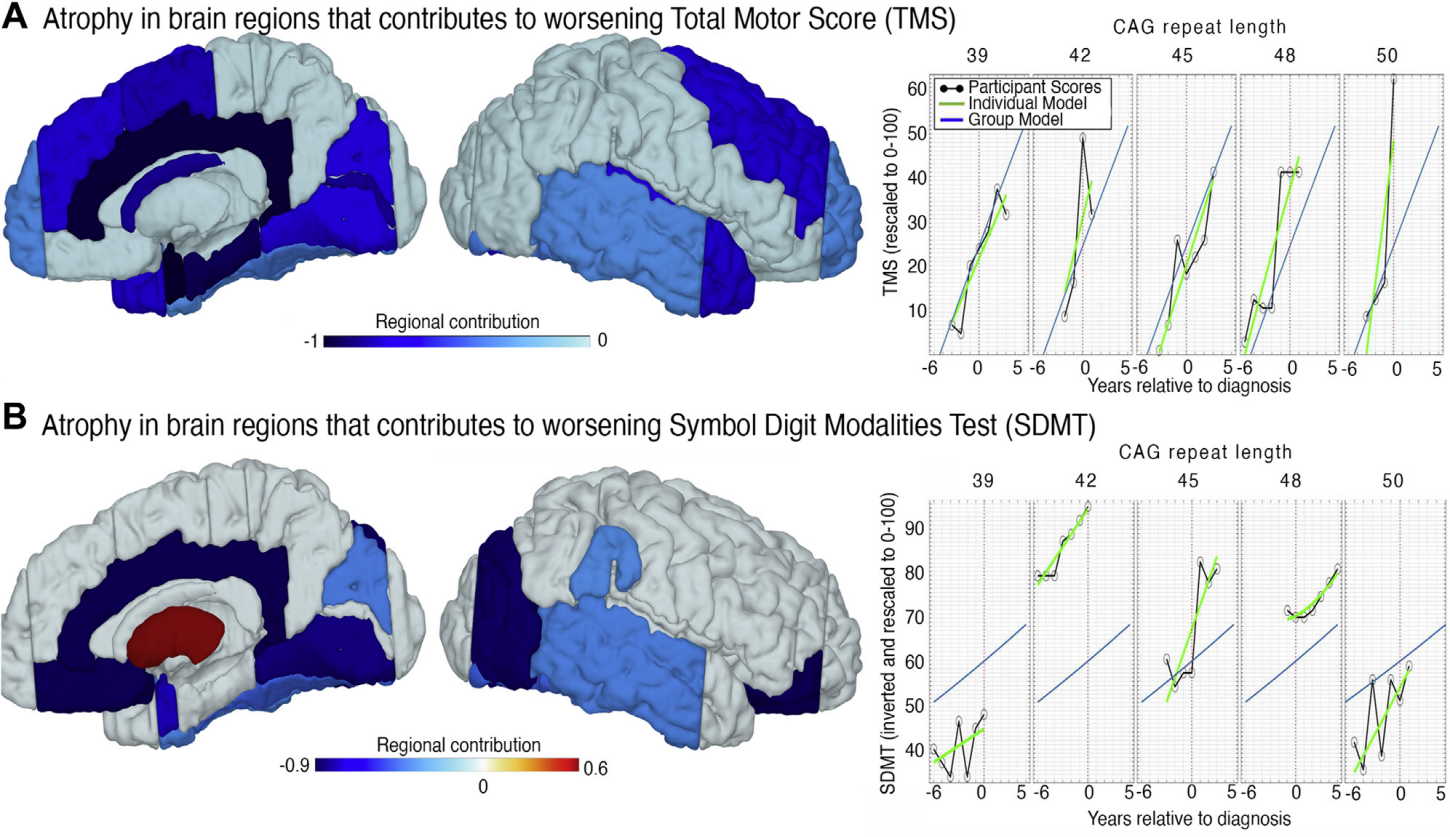
Brain-behavioral model predicting symptom changes during transition to Huntington’s disease (HD). **(A)** Surface projection of weights that indicate whether a brain region contributes to the prediction of longitudinal motor scores over all time points (for model illustration and details, see Figure S5 and the bar plot of weights in Figure S5B, C). Results are from group-level model accounting for effects of age, gender, CAG, and confounds (see Methods and Materials). The right panel illustrates the observed Unified Huntington’s Disease Rating Scale Total Motor Score (TMS) (scaled to 0–100, gray) and individual model predictions (green) for 5 exemplary participants with varying CAG length, group-level model predictions (blue) using our HD progression model. See Figure S6 for all HD participant plots. x-axis: disease progression time in years relative to individual motor diagnosis. **(B)** Analogous findings for brain-based prediction of cognitive deficits. Symbol Digit Modalities Test (SDMT) score, inverted and scaled 0–100. (Bar plot of weights in Figure S5.)

Finally, to evaluate how regional brain atrophy might contribute to emerging motor and cognitive symptoms, we extended our HD progression model to a longitudinal brain-behavioral framework (Figures S5A and S6; Supplemental Methods and Materials) including 1) brain volumes, 2) TMS motor assessments (29), and 3) cognitive symptoms evaluated using the SDMT (30) in HD participants only. Over a decade, TMS performance was reduced by 57.80%, and the SDMT by 16.78%, and the determination coefficient *R*^2^ was .82 for TMS and .89 for the SDMT, suggesting a strong model fit. In predicting individual TMS changes, atrophy in a number of regions contributed to worsening TMS; the entorhinal area, cingulate, parahippocampal gyrus, caudate, calcarine cortex, supplementary motor cortex, temporal pole, frontal gyrus, lingual gyrus, cuneus, and planum temporale were all predictors of worsening TMS (Figure 5A and Figure S6). When using the model to predict change in the SDMT, we found a pattern suggesting that the difference between cortical and striatal atrophy was predictive of cognitive worsening (Figure 5B). That is, in participants undergoing similar rates of putamen atrophy, those with particularly emphasized cortical atrophy in the cingulate, orbital gyrus, occipital gyrus, lingual gyrus, and entorhinal area experienced greater cognitive decline.

## Discussion

HD is a devastating neurological condition with a complex interplay of physiological, neuronal, and behavioral changes (2). Our study provides a novel characterization of long-term cortical atrophy during a critical period in HD progression—the onset of motor symptoms. Although previous studies have suggested that pathological changes occur cortically (4,17,44,45), they have provided little insight into the long-term trajectories and association with worsening symptomatology. The heterogeneity of cognitive and psychiatric symptoms many years before motor decline (46) suggests individual variability in pathology-related brain changes. Using a Bayesian dynamic modeling framework applied to a large multisite longitudinal sample of participants at the same disease stage, we have shown widespread cortical volume differences across the cortex in HD participants during clinically diagnosed onset of motor symptoms when compared with control subjects. Interestingly, our results indicate that in the period surrounding motor diagnosis the trajectory of volumetric change is variable across the cortical mantle, with the highest rates of cortical atrophy in the occipital regions.

There were differences across the cortex between the gene carriers at motor diagnosis and the control participants, with the occipital, frontal temporal, and parietal areas showing lower volume in HD. The occipital lobe showed the fastest rates of atrophy, with the parietal regions also showing significantly greater change. In contrast, the rates of changes in anterior and temporal regions showed smaller differences. In terms of clinical and behavioral markers, CAG-repeat length, the key marker of genetic burden and individual disease onset (2), was most predictive of the individual rate of volume decline within occipital regions. The entorhinal area, cingulate, and regions of the occipital, frontal, and temporal lobes were associated with worsening motor performance, where cognitive decline was associated with atrophy in the occipital, cingulate, and lingual regions. Our framework models regional brain alterations in relation to behavior and clinical changes rather than simply correlating cortical atrophy with symptoms across participants (17,47), and we present the first detailed picture of the process of cortical changes during disease onset and their direct effect on symptoms.

Although HD participants showed significantly lower volumes in frontal and temporal regions compared with control participants, these anterior regions did not show significantly greater rates of atrophy, suggesting that anterior atrophy occurs earlier in the disease course. Cognitive and psychiatric symptoms typically become apparent before motor symptoms, and studying the participants further from onset would explore the link between earlier emergence of these symptoms and frontotemporal atrophy.

Interestingly, during motor onset the cortical atrophy appears to contribute more to cognitive decline than that of the striatum. The SDMT, which measures psychomotor speed and visual processing, recruits widespread areas of frontoparietal and fronto-occipital networks along with temporoparietal and inferior frontal cortices (48). We observed an association between SDMT performance and atrophy progression in occipital regions, along with the cingulate and lingual gyrus, regions recruited during SDMT performance (49). Our results suggest that in participants showing similar rates of striatal atrophy, those with greater cortical atrophy in these regions might also undergo greater decline in SDMT performance. As such, cortical rather than striatal atrophy appears to be predictive of individual cognitive decline, with higher between-participant variability possibly due to unknown mediating factors resulting in partially independent progression trajectories of cortical and subcortical atrophy.

Conversely, we show that increased atrophy in a range of regions is associated with worsening motor scores. A number of regions, including the entorhinal area, cingulate, parahippocampal gyrus, caudate, calcarine cortex, supplementary motor cortex, lingual gyrus, and cuneus, are associated with spatial, motor, and visual performance. Given that TMS assesses motor behavior, eye movement, and clinical characteristics of HD, it is perhaps unsurprising that the TMS is associated with cortical regions linked to a range of functions.

Using our Bayesian dynamic modeling framework, we also explored between-region progression of cortical atrophy, but we found no evidence of it. It is likely that interregional interactions between cortical areas follow more complex processes than simple striatal-cortical or cortical-cortical spread, with other tissue types and variable time-lag factors playing a role. The integration of diffusion metrics into the model could help elucidate these processes. Alternatively, more power may be required. Future work will evaluate this theory by including multimodal and microstructural measures within the longitudinal modeling framework and investigate inter-region progression.

An additional strength of our approach is that it not only allows inference of group-level changes but also examines the contribution of genetic risk factors to individual differences in the progression of atrophy. CAG-repeat length showed a positive relationship with increased atrophy in subcortical and occipital regions, supporting a potential link between higher CAG-repeat length and HD progression (7,50), particularly within subcortical and occipital regions (15,51,52). Previous work had also demonstrated substantial occipital lobe atrophy in both pre-HD and manifest HD (4,6,15,53). The association between CAG-repeat length and occipital atrophy suggests that early visual regions are impacted by genetic burden more than other cortical regions, highlighting a differential relationship between cortical atrophy and genetic burden.

The ability of our modeling approach to detect subtle regional volume changes may make it an ideal model for analysis of clinical trial data in neurodegeneration. If a disease-modifying treatment were successful in changing the course of neurodegeneration, differences in neural atrophy between placebo and treatment groups could be anticipated. This model can be applied to any brain region and participant group, encouraging the application of our model to all neurodegenerative conditions. In HD, for example, the caudate, putamen, or sensory-motor regions could be measured to track effects of disease-modifying treatments (depending on the predicted treatment effects); in frontotemporal dementia, regions of the insula or temporal lobes could be selected instead (54).

The modeling framework used here was developed to address weaknesses in previous analysis methods and approach quantification of GM change via a dynamic systems method (23) in a unique longitudinal HD cohort. The ability to quantify changes in cortical GM atrophy over time, while accounting for individual variability over multiple time points, offers a more powerful approach than previous methods of structural MRI modeling (55,56). Indeed, the results of our leave-one-out cross-validation analysis indicate that our model is appropriate for modeling our data. However, it is important to consider that the use of clinical-rated motor diagnosis for temporal alignment of the progression of all participants in our model introduces some potential between-participant error because participants were seen yearly and could have converted at any point between two visits. Moreover, our focus on participants within 6 years of motor symptom onset prevented us from studying the very earliest cortical atrophy patterns. However, we carefully accounted for differences due to age, gender, total intracranial volume, and site to render the group inference unbiased. The regional progression observed during a particularly crucial period in HD progression is, therefore, clinically meaningful and is supported by previous imaging data, indicating that the model successfully illustrates longitudinal neurodegeneration in unprecedented depth.

In conclusion, our findings provide the most detailed characterization of cortical atrophy in HD presented to date. By applying a recently validated model that is uniquely able to map temporal and spatial cortical changes within a genetically confirmed HD cohort, we have demonstrated that cortical atrophy shows regional variability related to genetic factors and predicts motor and cognitive performance, representing changes within the HD phenotype. This work represents a principled approach to modeling longitudinal structural MRI data that offers new insights into the spatial and temporal phenotype of cortical changes and, in turn, the biological underpinnings of neurodegeneration.

## Supplementary Material

Supplementary Material

Key Resources Table
