# Erratum

**DOI:** 10.1016/j.biopsych.2021.05.020

**Published:** 2021-08-15

Erratum to: “Dynamics of Cortical Degeneration Over a Decade in Huntington’s Disease,” by Johnson *et al.* (*Biol Psychiatry* 2021; 89:807–816); https://doi.org/10.1016/j.biopsych.2020.11.009.

The authors have recognized an error in Figure 1, where the graph in panel C was mislabeled. Specifically, the order of the bar graphs was incorrect: Sigmoidal input; No input; Linear; Quadratic. Instead, the correct order is: No input; Sigmoidal input; Linear; Quadratic. The corrected version of this figure now appears in the paper.

