# Erratum

**DOI:** 10.1016/j.biopsych.2021.07.006

**Published:** 2021-10-01

Erratum to: “Dynamics of Cortical Degeneration Over a Decade in Huntington’s Disease,” by Johnson *et al.* (*Biol Psychiatry* 2021; 89:807–816); https://doi.org/10.1016/j.biopsych.2020.11.009.

The graph in panel C of Figure 1 was recently corrected via an erratum. At the same time that panel C was corrected, the y-axis on panel B was mistakenly edited from “rate of change” to “negative rate of change.” This change was not accurate and was also inadvertently not reported in the erratum. The figure has again been edited to correctly specify the y-axis label as “rate of change” in panel B.

In addition, in response to an issue raised by readers, the authors have added a scale marker of zero on the y-axis in this latest revision of the figure. Figure 1B aims to illustrate potential changes in the rate of change (i.e., nonlinearities) during disease progression. Since the state variable describes volume and because regions of interest lose volume during disease progression, the rate of change of volume is negative at all times. More specifically, an acceleration of the disease progression would be reflected by a decrease in the rate of change of volume over time, becoming even more negative. Notably, the implemented sigmoidal model flexibly enabled both directions, allowing for potential region-specific accelerations or decelerations of progression. However, the model comparison revealed highest evidence for the model without additional inputs (those that enabled changes in the rate of change in addition to self-connections).

The latest corrected version of this figure now appears in the paper.

