# Supplementary material for: Dynamics of Cortical Degeneration Over a Decade in Huntington’s Disease
Source: Biol Psychiatry. 2021 Apr 15;89(8):807–16. doi: 10.1016/j.biopsych.2020.11.009 (PMC7986936; doi:10.1016/j.biopsych.2020.11.009)
Supplement: Supplementary Material [file mmc1.pdf]

Dynamics of Cortical Degeneration Over a Decade in Huntington's Disease

Supplementary Information

Contents:

Supplementary Methods

Supplementary Figures S1-S6

Supplementary Table S1

Supplementary References

Supplementary Methods and Materials

Participants

For TRACK-HD, participants attended four yearly visits and were divided into control, Pre-HD and manifest HD groups at recruitment(1). TrackOn-HD followed on from the TRACK-HD study and included both a subset of TRACK-HD participants and newly recruited participants. For TRACK-HD, gene-positive participants were required to have a positive genetic test of ≥40 CAG repeats and a burden of pathology score > 250(2). Pre-HD participants all had a Total Motor Score (TMS) on the Unified Huntington's Disease Rating Scale (UHDRS)(3) of < 5 at baseline, indicating a lack of motor symptoms. Participants were classed as converters if they were recruited into the study as Pre-HD based on the above criteria, and then received a Diagnostic Confidence Score (DCS) score of ≥4 at any subsequent timepoint, indicating that they had met clinical diagnostic criteria for manifest HD. Fifty participants met this criterion for conversion during TRACK-HD/TrackOn-HD. One additional participant received a DCS of 4 at TRACK-HD visit 4, but then reverted to DCS <4 at a later timepoint and was excluded from this investigation.

Control participants for the TRACK-HD and TrackOn-HD studies were recruited age-matched and gender-matched to individuals in the combined preHD and HD groups and were selected from the spouses or partners of individuals with premanifest or early HD or were gene-negative siblings(1). For this analysis, a subset of 49 participants from the available pool of control data were included. These participants were selected to match the HD converters who were included in the final analysis as closely as possible on age, sex, site and number of scans.

Cognitive and motor performance

The UHDRS TMS was used to approximate clinical motor progression(3). TMS measures the presence of motor symptoms, and ranges from 0-60 with a score of <5 indicating no substantial motor symptoms. It requires participants to perform various motor tasks (for example, tandem walking) while being observed by a trained clinician, and shows high reliability(3).

The Symbol Digit Modalities Test (SDMT) assesses visuomotor integration and has components of visual scanning. It is a pencil-and-paper task during which participants view a key showing symbols with the digits 1-9. The participants are then presented with symbols

above an empty box and given 90 seconds to write the corresponding digits in the boxes. The SDMT is a reliable and key cognitive measure used in HD studies, and is also related to HD progression(4).

MRI data acquisition

Two scanners were Siemens and two were Philips. The parameters for Siemens were TR = 2200ms, TE = 2.2ms FOV = 28cm, matrix size = 256x256, 208. For Philips TR = 7.7ms, TE = 3.5ms, FOV = 24cm, matrix size = 242x224, 164. The acquisition was sagittal to cover the whole brain. There was a slice thickness of 1mm, with no gap between slices. These acquisition protocols were validated for multi-site use (1). Scanners were monitored over time to ensure consistent acquisition of images and all images were visually assessed for quality at time of data collection; specifically artefacts such as motion, distortion and poor tissue contrast (IXICO Ltd. and TRACK-HD imaging team, London, UK).

Longitudinal data processing

This process included between-timepoint scan registration, creation of an average participant scan for all timepoints and differential bias correction for between-timepoint scan inhomogeneity (5). Registration was performed using default settings and visual quality control (QC) was performed on all registered scans. Each average scan was then parcellated into 138 regions using MALP-EM, a fully automated segmentation tool (6) validated for use in HD (7); previously manually segmented whole-brain regions were binarized and included in the MALP-EM pipeline to improve brain extraction (8).

Bayesian hierarchical modelling for brain volume progression

Individual level dynamical model

The dynamical system used for modelling brain volume changes is generally described via state model $dx/dt=Ax+Cu$ and observational (or measurement) model introduced in the main methods. The endogenous dynamics Ax of the HD model were restricted to regional self-connections, i.e. A is a diagonal matrix. The diagonal elements can be interpreted as region-specific atrophy (or decay) rates $a_{ii} = a_0 e^{\lambda_i}$ (with $a_0 = -0.0025$; i.e. using log-normal priors for enforcing negativity), causing decay which results (for our value range) in approximately

linear volume loss over course of the progression. The assumed bilateral symmetry of the observational model (i.e. the same state variable describes the evolution of volumes in both corresponding bilateral ROIs) is based on previous structural MRI results reporting largely symmetric effects of atrophy in the TRACK-HD cohort and in a meta-analysis of HD studies (9–11) and is a common practice in Structural Equation Modelling(12).

The state equation $dx/dt=Ax+Cu(t)$ including external system inputs $u(t)$ determine the possible trajectories of state evolution during system integration. In this model inputs describe unknown underlying factors that can influence atrophy within a region on top of its endogenous decay. This can be used to implement explicitly defined external input factors that affect regions differentially through estimated input sensitivity (or amplitude) parameters C. More specifically, here we use the flexible framework to implement conventional progression models such as first and second order polynomials (a) & (b). Notably, for those the system is degenerate since $A=0$. Moreover, we use two dynamical models with diagonal matrix A, one without input ($C=0$), and one with actual sigmoidal input causing potential accelerations or decelerations (see below). The following state-equation models (illustrated in Figure 1B) were implemented:

(a) $dx/dt=c_0$, constant velocity model (i.e. volume follows linear function of time);

(b) $dx/dt=c_0+c_1t$, linear velocity model (i.e. volume follows quadratic polynomial)

(c) $dx/dt=Ax$, linear dynamical system without external inputs (C=0)

(d) $dx/dt=Ax+Cu_{sigmoid}$, linear system with sigmoidal input.

More specifically, to allow for a potential acceleration of disease pathology we studied sigmoidal inputs

$$Cu(t,\theta_u) = -c/(1+e^{-b(t-m)})$$

with regional amplitudes c, time-shifts m, and (always global) rate of change parameter b (illustrated in Figure 1B). The sensitivity C to (or amplitude of) the inputs indicates regional acceleration of shrinkage during progression ($c > 0$), decelerations ($c < 0$), or no contribution of the input ($c = 0$). The time-shift parameter of each regional sigmoid enabled earlier ($m < 0$) or later accelerations ($m > 0$) in years relative to motor diagnosis. Notably, the motivation for a sigmoidal model was due to previous reports pointing out that that analysis of the second-

derivative of imaging measurements revealed that AD-specific cortical thinning exhibited early acceleration followed by deceleration (13,14).

Bayesian model comparisons revealed highest model evidence for sigmoidal progression models without additional inputs (c) (Figure 1C), followed by model (d) and the polynomial models. All above first level model inversions were performed using previously established Variational Laplace methods[24]. More specifically, system integration and estimation followed a symmetric scheme from time point of motor diagnosis (treated as the initial volume state $x(t_0)$ of the system since data was available for all participants) both forward and backwards in time to the earliest time point prior to diagnosis and the latest time point after motor diagnosis. Since the volume data was standardized on the group-level the individual initial state $x(t_0)$ was estimates as additional parameters during model inversion and was compared across groups (Figure 3A).

Group-level model and covariates

The aim of the study is a group level disease progression model, fully accounting and potentially explaining individual level trajectories. All patients' first level models were embedded in a second (group-) level model. Here we took advantage of the recently introduced Parametric Empirical Bayes (PEB) framework for estimation and inference on multilevel non-linear models(15). As in Ziegler et al.(16), weakly informative priors were used for first level and second level parameters to allow results better reflect aspects of data rather than strong prior knowledge. Intercept, diagnosis group difference, and patient's subject-specific characteristics such as CAG repeat length, sex, age at motor diagnosis, total intracranial volume (TIV) and scanning site were included as covariates in the group level explaining first level variability.

The advantages of applying a Bayesian second level model to the full posterior of the individual subjects' parameters, rather than performing a classical analysis on the parameters' expected values (i.e., a CVA or MANCOVA) pertain to statistical efficiency and mitigating the risk of overfitting. Priors in a Bayesian model place constraints on the parameters, precluding implausible values (i.e., they serve the same role as regularization in classical statistics and machine learning). In the context of a hierarchical model, individual subjects' parameter

estimates are constrained by the group-level parameter estimates (empirical Bayes), reducing the risk of over-fitting in individual subjects. Furthermore, by conveying the full posterior density over parameters from the individual subjects to the group level, the effective degrees of freedom at the group level are correctly computed (whereas in classical statistics, this subject-level information is discarded). Most importantly, Bayesian methods facilitate efficient model comparison. The key idea is that in a (variational) Bayesian setting, model inversion and comparison are based on (an approximation of) the log model evidence, ln p(y|m). This scores the trade-off between the accuracy of the model and its complexity (the KL-divergence between the priors and posteriors), rather than just its accuracy. In the context of a hierarchical model, the overall complexity is the sum of the complexities at the within- and between-subject levels. Therefore, by inverting a full hierarchical model, the simplest explanation for the entire group's data is found that explains the most variance.

Notably, since CAG repeat length and age at diagnosis were found to be correlated highly (r=-0.85), age was entered after orthogonalization with respect to CAG. The hierarchical modelling (a) accounts for variation of first level parameters across participants; (b) explicitly allows assessing effects of e.g. CAG gene repeat length on all model parameters (e.g. decay rates); and (c) increases power for group level effects by accounting for first level parameter uncertainty differences across patients (e.g. scanned 3 vs. 7 times).

Bayesian Model Selection (BMS) was conducted comparing the obtained full hierarchical (two-level) models with above described first level forms and a second level design matrix including effects of interest, covariates and confounds. Bayesian model evidence accounts for both, optimizing model fit and while penalizing complexity and is therefore suitable for model selection in highly parameterized multilevel disease progression models(17).

In order to understand the role that CAG plays for the individual differences unfolding in those trajectories over the course of disease progression we used a post-hoc analysis to calculate the predicted variance (R^2) of volumes using CAG as a predictor for each time-point separately.

Model extensions to incorporate inter-regional interactions and symptoms

The generative disease progression model (illustrated in Fig. 1) was restricted to describe volumes in multiple brain regions only. Notably, this model did not allow for inter-regional dynamics i.e. the connection parameters were fixed to zero (diagonal matrix A). In order to test for potential inter-regional dynamics of regional morphometry during HD progression we explored various striatal-cortical and cortical-cortical networks (details and illustration in Supplementary Figure 4) using weakly informative priors on connection weights. However, highest model evidence was observed for uncoupled models (cf. model comparison in Supplementary Figure 4B) and thus all main findings were restricted to models with region-specific self-connections (diagonal of *A* matrix) and contribution of inputs (*C* matrix).

Finally, we extended the observational model (cf. section above) to additionally predict motor and cognitive symptom scores (TMS and SDMT) via regression using a linear combination of brain states:

$$y_{tms}(t) = w_0 + \sum_{i=1}^{28} w_i x_i(t) + \varepsilon$$

with regional weights w_i describing the contribution of each regional atrophy state to the prediction of the individual motor scores available for all brain scans in 49 participants (illustration in see Supplementary Figure 5). Notably, the comparably large number of observations per subject in this exceptional dataset allows assessing the association of brain states and motor scores over timepoints within-subject, in contrast to many conventional between-subject brain-behavioural findings.

p-values and Bayesian posteriors

In classical statistics, a p-value is the probability of finding an effect at least as large at that observed under the null hypothesis. The null hypothesis itself cannot be accepted. By contrast, in Bayesian statistics, the null hypothesis is just one of potentially many hypotheses, and the posterior probability for each hypothesis given the model and data is estimated. The model with highest probability given the data was chosen and presented. The posterior distributions of parameters given the data under the highest evidence model are presented in the main results.

There is no formal equivalence between p-values and posterior probabilities (see Lindley's paradox - Lindley, 1957).

Data and code availability

Requests for access to the TRACK-HD and TrackOn-HD data should be made via the CHDI Foundation. The link to custom made scripts and synthetic example dataset that demonstrate SPM-based dynamic modelling of longitudinal HD data applied during this study can be provided upon request to the corresponding authors. Please open readme.txt for further details on how to apply the code. Please note that this particular code has not been thoroughly tested with other software versions than MATLAB2020a, SPM12 r7771 and other parameter choices might produce errors during processing attempts. The code aims at transparency and illustration but is not intended for clinical use. It is free but copyright software, distributed under the terms of the GNU General Public License as published by the Free Software Foundation (either version 2, or at your option, any later version). Further details on "copyleft" can be found at http://www.gnu.org/copyleft/. In particular, software is supplied as is. No formal support or maintenance is provided or implied. For any questions and requests please contact gabriel.ziegler@dzne.de.

Supplementary Figures

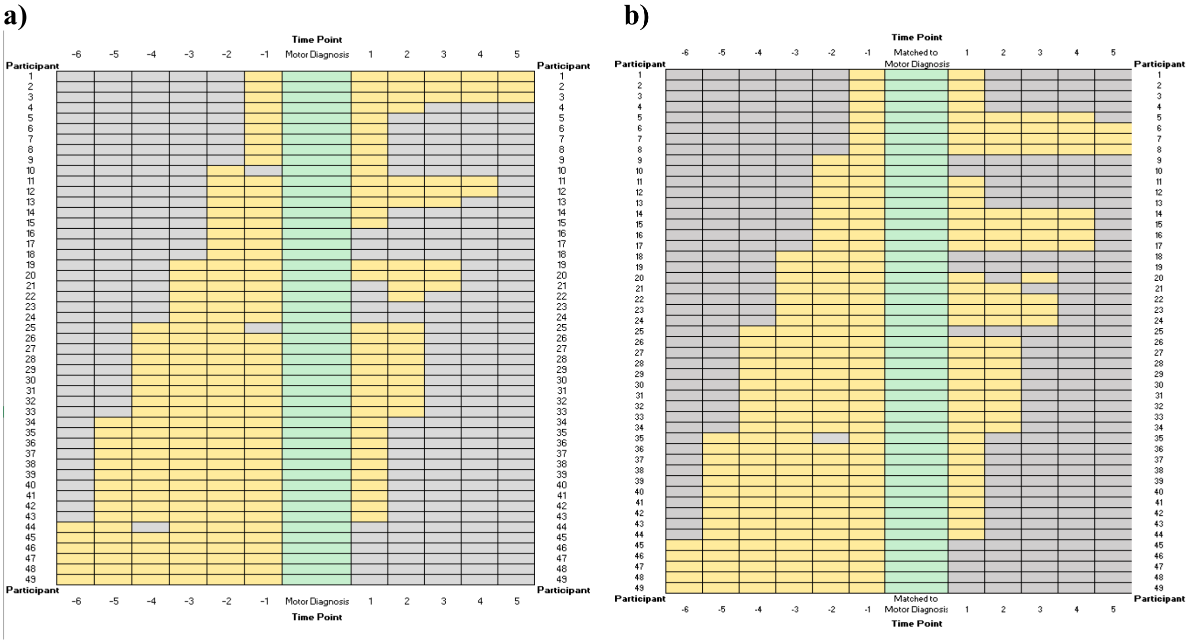

Supplementary Figure S1. **A schematic showing all MRI scans included in analysis.** a) showing HD participants and b) showing matched control participants. Data is presented re-aligned to motor diagnosis across participants. Green represents motor diagnosis, yellow represents available MRI data and grey represents missing data. Missing data includes time-points for which a participant was not yet recruited (e.g. when a participant was recruited at baseline of TrackOn-HD, such as Participant 49), or had dropped out of the study (e.g. Participant 16 dropped out at the end of TRACK-HD and did not participate in TrackOn-HD), and when a participant could not attend a time-point (e.g. Participant 26). X-axis: disease progression time (in years relative to individual motor diagnosis) also used for dynamic HD modelling.

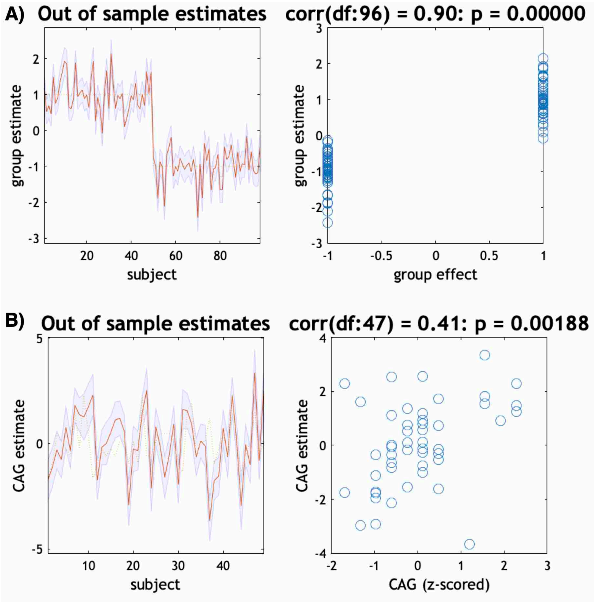

Supplementary Figure S2. Validation of the dynamical disease progression model. Out of sample predictions using Leave one out (LOO) cross-validation. **(A)** Predictions of individual group membership (1 HD, -1 NO) using group model parameters after training the PEB model using all other subjects of the sample (N=98-1, left panel). Right panel shows the group membership estimated over the actual group membership. Y-axis: Estimates (±SD) of second level group variable. X-axis: Number of subject predicted. **(B)** Prediction of individual CAG length using patient model parameters after training the PEB model using all other participants (N=49-1, left panel). Right panel shows predicted CAG over actual CAG of the participant.

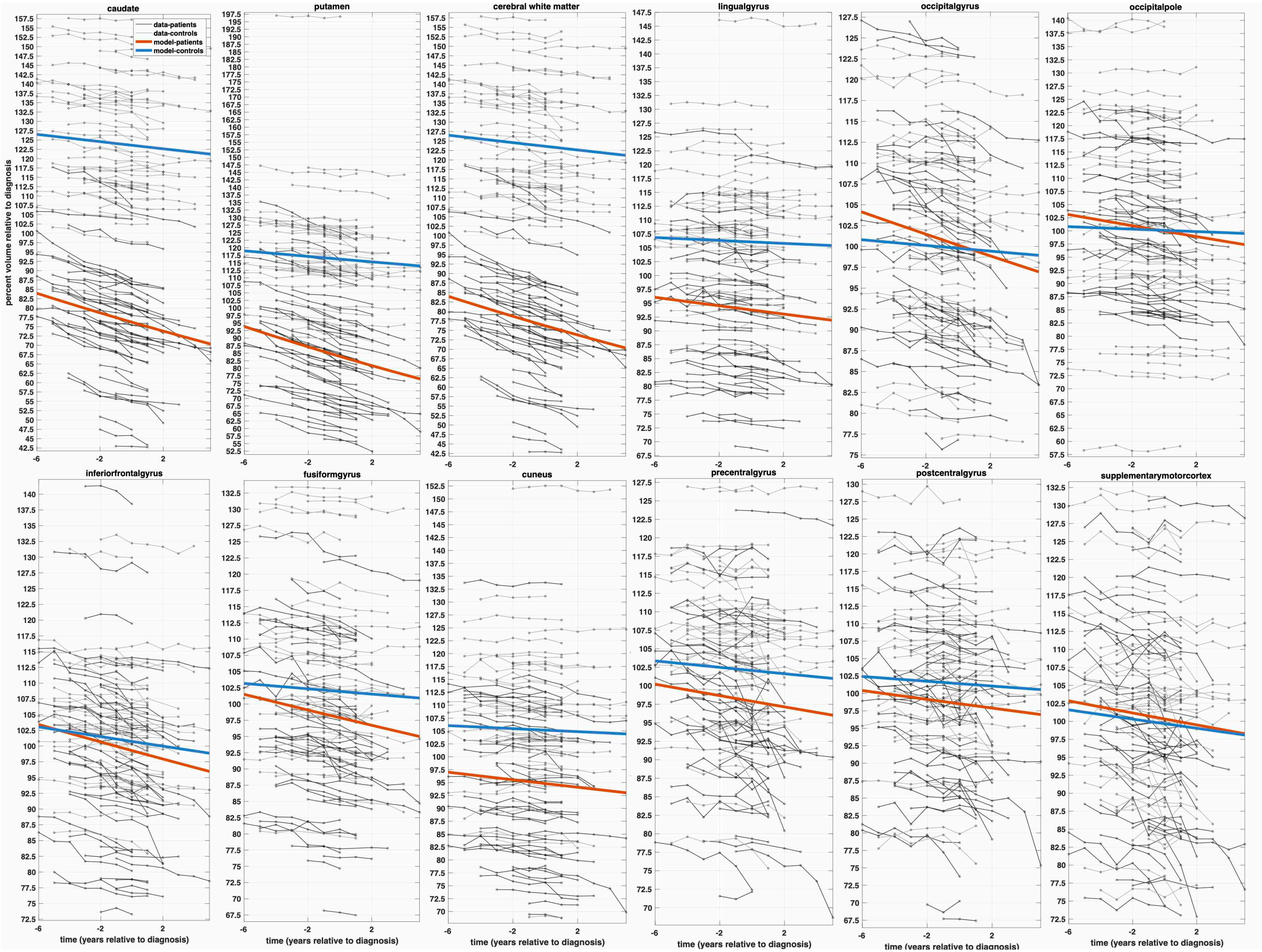
caudate
putamen
cerebral white matter
lingualgyrus
occipitalgyrus
occipitalpole
inferiorfrontalgyrus
fusiformgyrus
cuneus
precentralgyrus
postcentralgyrus
supplementarymotorcortex
data-patients
data-controls
model-patients
model-controls
percent volume relative to diagnosis
time (years relative to diagnosis)

Supplementary Figure S3. Regional volume data from selected ROIs and model predictions of the HD progression model. Plots show regional longitudinal raw data (percent volume relative to volume at year of motor diagnosis) with each thin black line representing one of the 98 participants. The group level predictions for HD participants from our progression model with highest Bayesian model evidence is shown in red and healthy age-matched controls in blue. With exception of the white matter volume, regional volumes refer to the left hemisphere with corresponding right hemispheric volume exhibiting very similar progression (not shown). Y-axis: percent volume (relative to mean volume of whole group at time-point of diagnosis). X-axis: disease progression time in years relative to individual motor diagnosis. For more details on trajectory estimation see methods and supplementary notes on modelling.

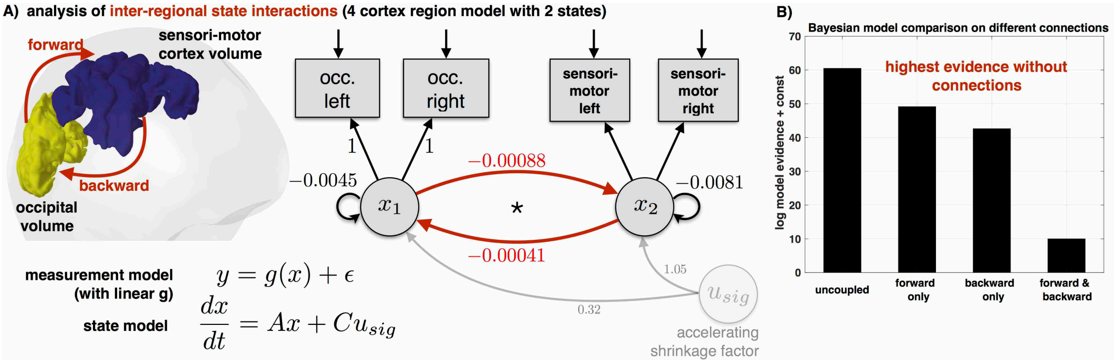

Supplementary Figure S4. Exploring inter-regional dynamics during HD progression. We explored potential disease spreading including regional connections in the dynamical system (state model with connectivity matrix *A,* cf. supplementary notes on modelling). We particularly focussed on striatal-cortical (not shown) and cortical-cortical interactions. **(A)** We hypothesized and tested interactions by focussing on 2 (bilateral) region models with occipital and sensory-motor cortex volume respectively (top left surface projection). Off-diagonal elements of connectivity matrix were used to test whether individual volume state in region 1 (occ.) significantly affects rates of change of progression in region 2 (sensory-motor cortex), above and beyond decay induced by self-connections (i.e. rate of atrophy/decay). Forward, backward and bi-directional connectivities were implemented and included as separate models in the model space. **(B)** Bayesian model comparison revealed highest model evidence for models without additional inter-regional connections. This suggests that spatial and temporal dynamics of volume changes are parsimoniously described using local dynamical parameters and inputs presented in main results. Neuronal processes such as micro-structure (not optimally) reflected in macroscopic volumes might be involved in causal unfolding of HD disease pathology (cf. methods).

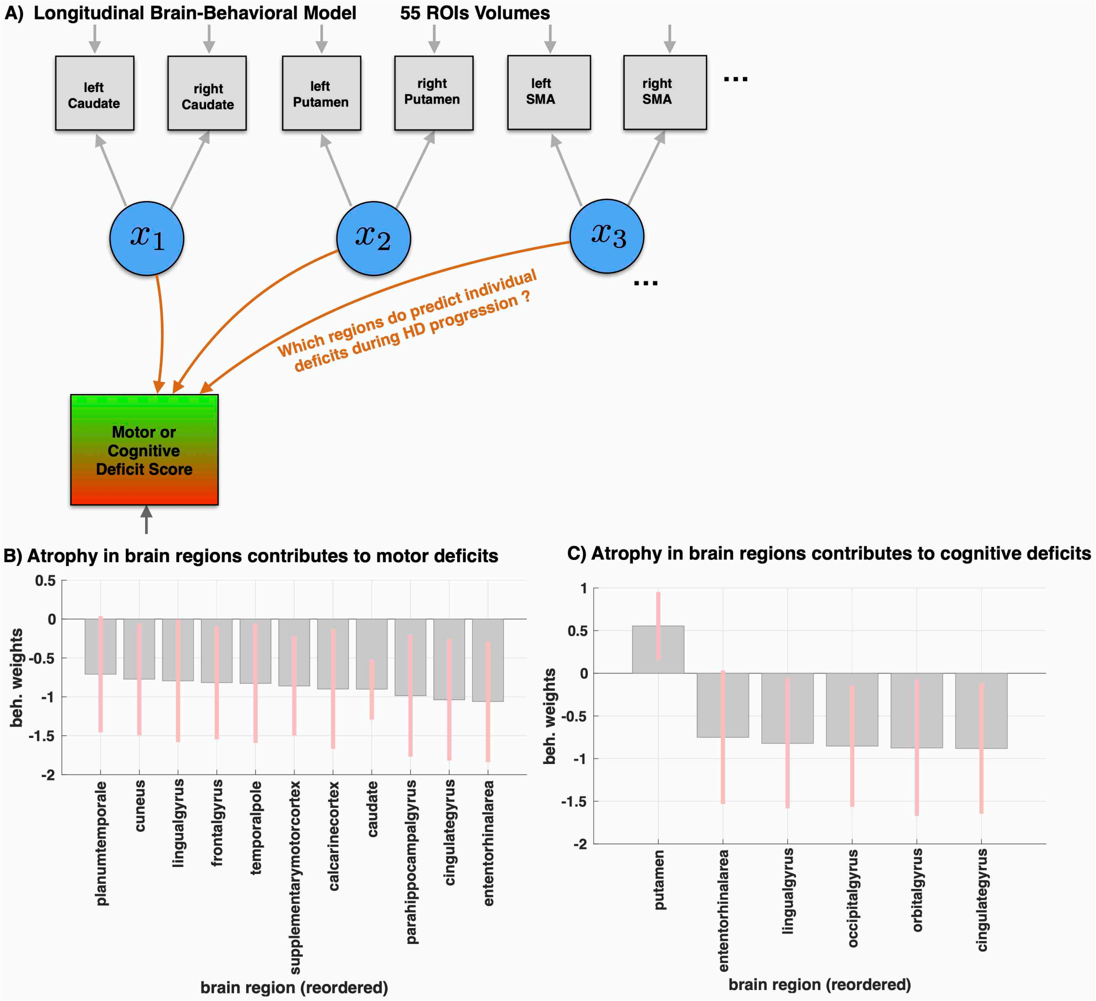

Supplementary Figure S5. Extending the dynamic model to predict individual symptom changes during transition towards HD. (A) Illustration of an extended brain-behavioural dynamical model predicting longitudinal observations from two domains, i.e. regional brain volumes (grey boxes) and TMS motor scores (red-green box). The 28 hidden brain states *x(t)* (blue circles) inferred in the generative model were here used to simultaneously predict longitudinal TMS motor scores available for all time-points with MRI scans from 47 of 49 HD patients. Notably, full model inversion is performed jointly with brain and behavioural data. The TMS prediction is following a multiple linear regression via an extended observational model (linear combination of states with prior weights around zero, cf. methods). Larger weights in a region indicate a higher contribution of the (time-varying) regional atrophy to prediction of symptoms during progression. (B) Here we show additional bar plots of significant prediction weights (mean ± SD, illustrated in Figure 5) when predicting motor symptoms (using the Bayesian Model Reduction and Averaging). Examples of 5 individual participant model predictions for TMS scores are shown in Figure 5 (also in Supplementary Figure 6). (C) Here we show bar plots of prediction weight parameters (mean ± SD, illustrated in Figure 5) when predicting cognitive symptoms.

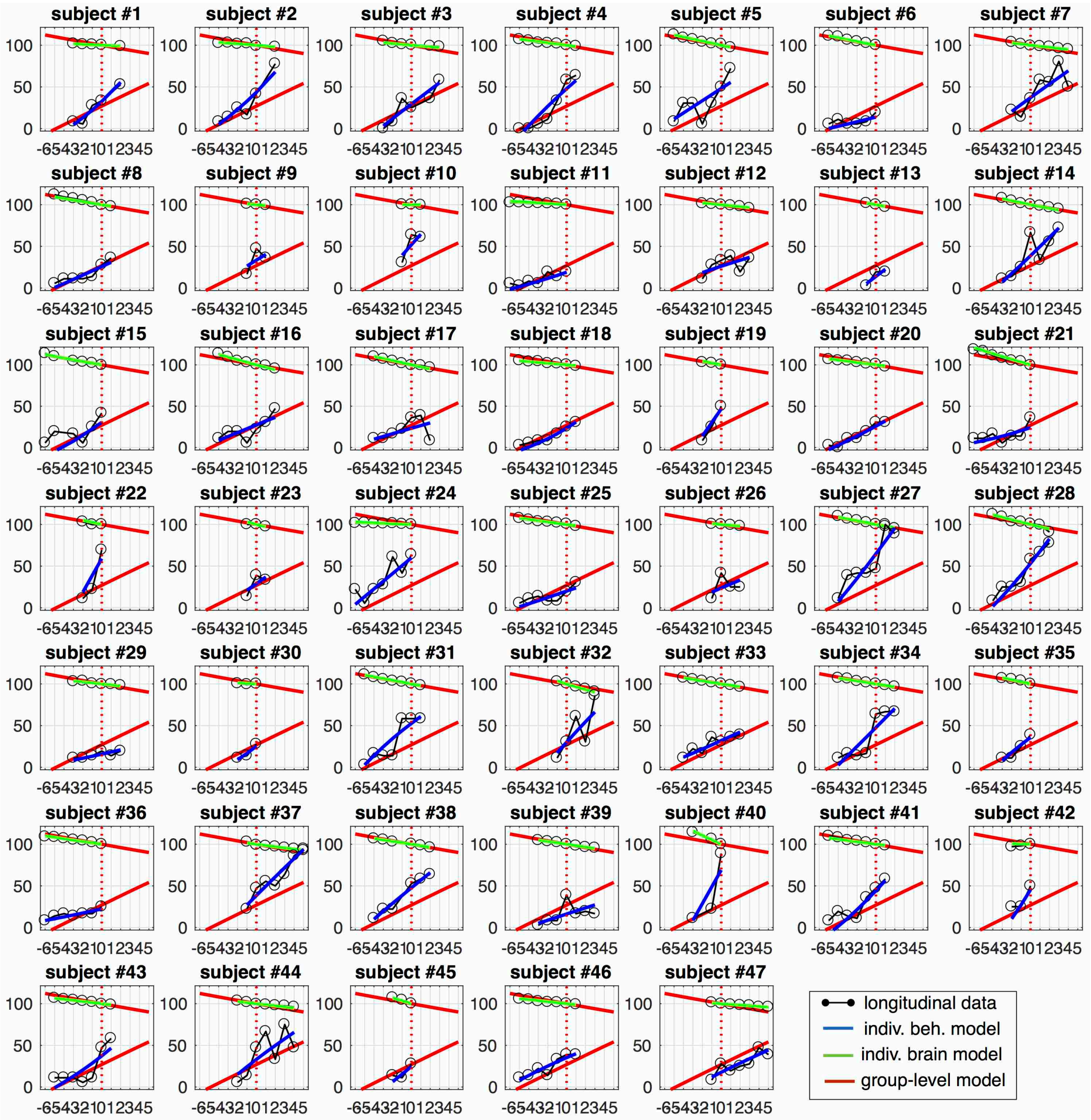

Supplementary Figure S6. Example of individual-level model of caudate and motor score disease progression trajectories. The main generative model predicts individual regional brain volumes during HD disease progression. The upper part of the plot shows the individual-level (green) as well as group-level (red) model predictions and the observed caudate volumes (black, % relative to motor diagnosis) for 47 of 49 subjects with available MRI scans and motor symptom assessments (TMS). We also extended the model to a generative brain-behavioral model including motor symptoms (cf. parameters shown in Figure 5A and Supplementary Figure 5, cf. methods). Lower part of the plot shows the TMS observations (black, normalized to [0, 100]) and corresponding individual-level (blue) and group level model predictions (red) of all 47 subjects with available data. Y-double-axis: percent volume (relative to volume at time-point of diagnosis) or TMS scaled to 0-100. X-axis: disease progression time in years relative to individual motor diagnosis.

Supplementary Table S1. The final brain regions measured in this study, and the original regions output by MALP-EM that were combined to create the final regions.

Final Region Name	Original Combined Regions
Angular Gyrus Right	Angular Gyrus Right
Angular Gyrus Left	Angular Gyrus Left
Calcarine Cortex Right	Calcarine Cortex Right
Calcarine Cortex Left	Calcarine Cortex Left
Cuneus Right	Cuneus Right
Cuneus Left	Cuneus Left
Entorhinal Area Right	Entorhinal Area Right
Entorhinal Area Left	Entorhinal Area Left
Frontal Pole Right Occipital	Frontal Pole Right
Frontal Pole Left	Frontal Pole Left
Lingual Gyrus Right	Lingual Gyrus Right
Lingual Gyrus Left	Lingual Gyrus Left
Occipital Pole Right	Occipital Pole Right
Occipital Pole Left	Occipital Pole Left
Precuneus Right	Precuneus Right
Precuneus Left	Precuneus Left
Parahippocampal Gyrus Right	Parahippocampal Gyrus Right
Parahippocampal Gyrus Left	Parahippocampal Gyrus Left
Planumtemporale Right	Planumtemporale Right
Planumtemporale Left	Planumtemporale Left
Supplementary Motor Cortex Right	Supplementary Motor Cortex Right
Supplementary Motor Cortex Left	Supplementary Motor Cortex Left
Supramarginal Gyrus Right	Supramarginal Gyrus Right
Supramarginal Gyrus Left	Supramarginal Gyrus Left
Superior Parietal Lobule Right	Superior parietal Lobule Right
Superior Parietal Lobule Left	Superior parietal Lobule Left
Temporal Pole Right	Temporal Pole Right
Temporal Pole Left	Temporal Pole Left
Temporal Gyrus Right	Right Inferior Temporal Gyrus; Right Medial Temporal Gyrus; Right Planum Polar; Right Superior Temporal Gyrus; Right Transverse Temporal Gyrus
Temporal Gyrus Left	Left Inferior Temporal Gyrus; Left Medial Temporal Gyrus; Left Planum Polar; Left Superior Temporal Gyrus; Left Transverse Temporal Gyrus
Orbital Gyrus Right	Right Anteriororbital Gyrus; Right Gyrus Rectus; Right Lateral Orbital Gyrus; Right Medial Frontal Cortex; Right Medial Orbital Gyrus; Right Posterior Orbital Gyrus; Right Subcolossal Area

**Final Region Name**	**Original Combined Regions**
Orbital Gyrus Left	Left Anteriororbital Gyrus; Left Gyrus Rectus; Left Lateral Orbital Gyrus; Left Medial Frontal Cortex; Left Medial Orbital Gyrus; Left Posterior Orbital Gyrus; Left Subcolossal Area
Cingulate Gyrus Right	Right Anterior Cingulate Gyrus; Right Middle Cingulate Gyrus; Right Posterior Cingulate Gyrus
Cingulate Gyrus Left	Left Anterior Cingulate Gyrus; Left Middle Cingulate Gyrus; Left Posterior Cingulate Gyrus
Frontal Gyrus Right	Right Superior Frontal Gyrus; Right Superior Frontal Gyrus Medial Segment; Middle Frontal Gyrus
Frontal Gyrus Left	Left Superior Frontal Gyrus; Left Superior Frontal Gyrus Medial Segment; Middle Frontal Gyrus
Occipital Gyrus Right	Right Superior Occipital Gyrus; Right Inferior Occipital Gyrus; Right Middle Occipital Gyrus
Occipital Gyrus Left	Left Superior Occipital Gyrus; Left Inferior Occipital Gyrus; Left Middle OccipitalGyrus
Inferior Frontal Gyrus Right	Right Tringular Part Of The Inferior Frontal Gyrus; Right Orbital Part Of The Inferior Frontal Gyrus; Right Opercular Part Of The Inferior Frontal Gyrus
Inferior Frontal Gyrus Left	Left Tringular Part Of The Inferior Frontal Gyrus; Left Orbital Part Of The Inferior Frontal Gyrus; Left Opercular Part Of The Inferior Frontal Gyrus
Operculum Right	Right Central Operculum; Right Frontal Operculum; Right Parietal Operculum
Operculum Left	Left Central Operculum; Left Frontal Operculum; Left Parietal Operculum
Insula Right	Right Posterior Insular; Right Anterior Insula
Insula Left	Left Posterior Insular; Left Anterior Insula
Postcentral Gyrus Right	Post Central Gyrus Right; Right Postcentral Gyrus Medial Segment
Postcentral Gyrus Left	Post Central Gyrus Left; Left Postcentral Gyrus Medial Segment
Precentral Gyrus Right	Precentral Gyrus Right; Right Precentral Gyrus Medial Segment
Precentral Gyrus Left	Precentral Gyrus Left; Left Precentral Gyrus Medial Segment
Fusiform Gyrus Right	Right Fusiform; Right Occipital Fusiform Gyrus
Fusiform Gyrus Left	Left Fusiform; Left Occipital Fusiform Gyrus

Supplementary References

1. Tabrizi SJ, Langbehn DR, Leavitt BR, Roos RA, Durr A, Craufurd D, *et al.* (2009): Biological and clinical manifestations of Huntington's disease in the longitudinal TRACK-HD study: cross-sectional analysis of baseline data. *Lancet Neurol* 8: 791–801.

2. Penney JB, Vonsattel JP, MacDonald ME, Gusella JF, Myers RH (1997): CAG repeat number governs the development rate of pathology in Huntington's disease. *Ann Neurol* 41: 689–92.

3. Huntington Study Group (1996): Unified Huntington's Disease Rating Scale: reliability and consistency. *Mov Disord* 11: 136–42.

4. Tabrizi SJ, Scahill RI, Owen G, Durr A, Leavitt BR, Roos RA, *et al.* (2013): Predictors of phenotypic progression and disease onset in premanifest and early-stage Huntington's disease in the TRACK-HD study: analysis of 36-month observational data. *Lancet Neurol* 12: 637–649.

5. Ashburner J, Ridgway GR (2012): Symmetric diffeomorphic modeling of longitudinal structural MRI. *Front Neurosci* 6: 197.

6. Ledig C, Heckemann RA, Hammers A, Lopez JC, Newcombe VFJ, Makropoulos A, *et al.* (2015): Robust whole-brain segmentation: application to traumatic brain injury. *Med Image Anal* 21: 40–58.

7. Johnson EB, Gregory S, Johnson HJ, Durr A, Leavitt BR, Roos RA, *et al.* (2017): Recommendations for the Use of Automated Gray Matter Segmentation Tools: Evidence from Huntington's Disease. *Front Neurol* 8: 519.

8. Freeborough PA, Fox NC, Kitney RI (1997): Interactive algorithms for the segmentation and quantitation of 3-D MRI brain scans. *Comput Programs Biomed* 53: 15–25.

9. Tabrizi SJ, Reilmann R, Roos R, Durr A, Leavitt B, Owen G, *et al.* (2012): Potential endpoints for clinical trials in premanifest and early Huntington's disease in the TRACK-HD study: analysis of 24 month observational data. *Lancet Neurol* 11: 42–53.

10. Minkova L, Habich A, Peter J, Kaller CP, Eickhoff SB, Klöppel S (2017): Gray matter asymmetries in aging and neurodegeneration: A review and meta-analysis. *Hum Brain Mapp* 38: 5890–5904.

11. Minkova L, Gregory S, Scahill RI, Abdulkadir A, Kaller CP, Peter J, *et al.* (2018): Cross-sectional and longitudinal voxel-based grey matter asymmetries in Huntington's disease. *NeuroImage Clin* 17: 312–324.

12. Kievit RA, Brandmaier AM, Ziegler G, van Harmelen AL, de Mooij SMM, Moutoussis M, *et al.* (2018): Developmental cognitive neuroscience using latent change score models: A tutorial and applications. *Developmental Cognitive Neuroscience*, vol. 33. pp 99–117.

13. Sabuncu MR, Desikan RS, Sepulcre J, Yeo BTT, Liu H, Schmansky NJ, *et al.* (2011): The dynamics of cortical and hippocampal atrophy in Alzheimer disease. *Arch Neurol*. https://doi.org/10.1001/archneurol.2011.167

14. Oxtoby NP, Alexander DC (2017): Imaging plus X: Multimodal models of neurodegenerative disease. *Current Opinion in Neurology*. https://doi.org/10.1097/WCO.0000000000000460

15. Zeidman P, Jafarian A, Seghier ML, Litvak V, Cagnan H, Price CJ, Friston KJ (2019): A guide to group effective connectivity analysis, part 2: Second level analysis with PEB. *Neuroimage*. https://doi.org/10.1016/j.neuroimage.2019.06.032

16. Ziegler G, Ridgway GR, Blakemore S-J, Ashburner J, Penny W (2017): Multivariate dynamical modelling of structural change during development. *Neuroimage* 147: 746–762.

17. Penny WD (2012): Comparing dynamic causal models using AIC, BIC and free energy. *Neuroimage* 59: 319–330.
